# Changes in QTc interval in long-term hemodialysis patients

**DOI:** 10.1371/journal.pone.0209297

**Published:** 2019-01-03

**Authors:** Yoshihiro Matsumoto, Yasuo Mori, Shinji Kageyama, Kazuo Arihara, Hidemaro Sato, Kijun Nagata, Yasushi Shimada, Yohichi Nojima, Koichiro Iguchi, Toshikazu Sugiyama

**Affiliations:** 1 Department of Nephrology and Hemodialysis, Shizuoka City Hospital, Shizuoka, Japan; 2 Shibukawa Clinic, Shizuoka, Japan; 3 Kageyama Clinic, Shizuoka, Japan; 4 Ohtemachi Clinic, Shizuoka, Japan; 5 Sawada Hospital, Gifu, Japan; 6 Sugiyama Clinic, Shizuoka, Japan; International University of Health and Welfare, School of Medicine, JAPAN

## Abstract

**Background:**

Cardiovascular diseases, including sudden cardiac death (SCD), are the leading cause of death in hemodialysis (HD) patients. A prolonged QT interval on the electrocardiogram (ECG) is a risk factor for SCD in HD patients. This study investigated whether the heart rate-corrected QT (QTc) interval becomes prolonged along with dialysis vintage.

**Methods:**

A total of 102 HD patients were retrospectively studied. Their ECG data were analyzed at 1, 4, and 7 years after HD initiation. The control group comprised 68 age-matched individuals who had normal renal function and two available ECG reports at an interval of more than 4 years. QTc was measured according to the Bazett formula. The association between QTc interval and dialysis vintage was studied. Additionally, clinically relevant variables related to QTc duration at 1 year after HD initiation were assessed.

**Results:**

Average QTc interval at 4 and 7 years after HD initiation was significantly longer than that at 1 year after HD initiation (443, 445, and 437 ms) (p<0.05). On the other hand, QTc interval in the control group was 425 ms in the first year and 426 ms after an average of 6 years. They had no significant differences, although they were much shorter than that in HD patients. Multivariate regression analysis of baseline variables revealed that the corrected calcium levels (*p* = 0.041) and diabetes (*p* = 0.043) were independently associated with longer QTc interval.

**Conclusions:**

The QTc interval at 1 year after HD initiation was longer than in the control subjects and was prolonged over several years of HD treatment. Providing clinical management with a focus on QTc interval may be helpful for reducing the incidence of SCD in HD patients.

## Introduction

Patients with end-stage renal disease (ESRD) undergoing dialysis are at a particularly high risk of cardiovascular disease (CVD). Sudden cardiac death (SCD) is the most common cause of mortality in these patients, accounting for >25% of all deaths in United States [1]. Unlike in the general population, coronary artery disease is not a major cause of SCD in dialysis patients. Study of Heart and Renal Protection concluded that lowering LDL cholesterol with statin and ezetimibe did not result in decreased coronary mortality in patients with chronic kidney disease including dialysis patients [2]. A retrospective study reported that 71% of dialysis patients who died of SCD had either normal left ventricular (LV) function or mild-to-moderate dysfunction [3]. A small study using an implantable cardiac monitor in stable hemodialysis (HD) patients with more than 35% of LV function revealed that at least 4 of 5 SCD events occurred with severe bradycardia and ensuring asystole [4]. Neither ventricular arrhythmias nor QRS widening was recorded at the time of these deaths [4]. Therefore, it is hypothesized that dynamic electrical changes as well as profound myocardial alterations associated with metabolic and structural disorders are of greater importance in HD patients.

A prolonged QT interval on the electrocardiogram (ECG) usually indicates delayed ventricular myocardial repolarization. Previous reports have shown that abnormal heart rate-corrected QT (QTc) prolongation is an independent risk factor for SCD in patients with heart failure [5] and in the general older population [6], and that prolonged QTc interval predicts mortality in patients with Takotsubo cardiomayopathy [7] or type 1 diabetes [8]. Recently, Genovesi et al demonstrated that prolonged QTc was associated with SCD and total mortality in a case series of HD patients [9].

Previously, HD sessions have been shown to induce a progressive increase in QT interval [10]. *In vivo* and *in silico* analysis clearly revealed that the change of serum concentrations of calcium and potassium alter the ventricular repolarization duration, which depends on the level of these ions in the dialysate [11]. These effects may predispose some patients to ventricular arrhythmia during and after HD. On the other hand, it was reported that QTc interval before HD session in HD patients was longer than that in healthy controls [12]. Also, QTc interval was found to increase with each subsequent stage of chronic kidney disease (CKD) [13]. These results suggest that both repeated HD sessions and ESRD per se may promote prolongation of QT interval.

In the present study, changes in QTc interval after HD initiation in ESRD patients was investigated, and clinically relevant variables related to basal QTc duration in patients on HD were also assessed.

## Materials and methods

### Patient selection

Patients were recruited from 7 outpatient hemodialysis facilities. Eligibility criteria were determined by reviewing the medical records and ECG data. The patients who were 25 years or older at the time of HD initiation and had been undergoing 4-hour long HD, 3 times a week for at least 7 years, and whose ECG reports at 1, 4, and 7 years after HD initiation were available, were included in the study. We did not collect the data of QTc interval just before and after HD initiation because of the possibility of severe disturbance of electrolyte levels. They received standard medications such as vitamin D, phosphate binders, antihypertensive agents, and erythropoietin. Dialysate bathes contained 2.0 mEq/L K+, 2.5–3.0 mEq/L Ca++, and 1.0 mEq/L Mg++.

To create the comparison cohort, age-matched patients who were being followed up for management of diabetes mellitus, hypertension, hyperlipidemia or hyperuricemia, or who had been undergoing routine health checks, were identified at Shibukawa Clinic (Shizuoka, Japan). The patients who had at least two ECG reports at an interval of more than 4 years, were aged 40 years or over at the time of early ECG examination, and had normal renal function at the time of last ECG examination, were included in the control group.

In both HD group and control group, any patient who had heart rate <57 beats per minute (bpm) or >103 bpm, any rhythm other than sinus, or any instances of extrasystoles in their ECG reports were excluded from the study.

This study was approved by Ethics Committee of Shibukawa Clinic and all participants provided written informed consent for the study according to the Declaration of Helsinki.

### Baseline data collection at 1 year after HD initiation in patients with ESRD

In all the 7 HD facilities, pre- and post-dialysis blood samples were routinely collected every month, and ECG and chest X-ray were examined before HD session on the same day as blood tests at least every 3 to 6 months. From the medical records on the day when ECG examination was undertaken about one year after HD initiation, the following hematochemical variables were obtained: hemoglobin (g/dl), albumin (g/dl), urea (mg/dl), total calcium (mg/dl), phosphorus (mg/dl), potassium (mEq/l), and glucose (mg/dl). Albumin-corrected serum calcium was calculated using the following equation: corrected calcium (mg/dl) = [(0.8 x (4- measured serum albumin (g/dl)) + measured total calcium (mg/dl)] [14]. Dialysate calcium concentration, pre-dialysis body weight, blood pressure, cardiothoracic ratio (%) on chest X-ray, and data of medications used (angiotensin converting enzyme inhibitors (ACEIs), angiotensin receptor blockers (ARBs), Ca-blockers, beta-blockers, and antiarrhythmic medication), were also obtained. Medical histories such as cardiac surgery, percutaneous coronary intervention, and implantation of a pacemaker or a defibrillator until that day were collected.

### Evaluation of QTc interval in ECGs

The patients in the HD and control groups underwent 12-lead ECG at rest using ECG machines from Fukuda Denshi (Tokyo, Japan) or Nihon Kohden (Tokyo, Japan). For HD patients, it was done before HD session. The QT and RR intervals were measured based on the automated algorithms. The two companies used different programs to measure QT interval and different methods for calculation of QTc interval (Fukuda Denshi, Bazett formula; Nihon Kohden, ECAPS12 formula). To unify formulas to obtain QTc interval, we recalculated the results from Nihon Kohden machines using Bazett formula (QTc = QT/√RR), based on the corresponding QT and RR intervals. ECGs showing heart rate <57 bpm or >103 bpm, were excluded to improve the accuracy of correction of the QTc interval using Bazett formula.

### Statistical analyses

Data were expressed as percentage for categorical variables or as means and standard deviations for continuous variables. All analyses were conducted using DANS version 7.1 (Sugimoto Data Analysis Service, Nagoya, Japan).

Univariate analysis was used to identify the factors related to QTc interval at 1 year after HD initiation. Unpaired t-test or one-way layout analysis of variance was applied for categorical variables and linear regression analysis was applied for continuous variables. Subsequently, multivariate regression analysis was performed for selected variables which were significantly associated with the basal QTc interval at univariate analysis. A *p* value was considered statistically significant when <0.10 in the univariate analysis and when <0.05 in the multivariate analysis.

To compare the QTc interval between the two sets (at first year and after 6 years in the control group; at first year in the control group and at 1 year after HD initiation in the HD group), Student’s *t* test was used. To compare the QTc interval at 1 year, 4 years, and 7 years after HD initiation in the HD group, Dunett type multiple comparison was applied. A *p* value of <0.05 was considered statistically significant for both comparisons.

## Results

One hundred and two patients of HD (66 men, 36 women) and 68 control patients (27 men, 41 women) participated in the study. Clinical and demographic characteristics of HD group at 1 year after HD initiation are described in Table 1. The mean ages at 1 year after HD initiation in the HD group and at the time of first ECG analysis in the control group were 58.5 and 58.0 years, respectively.

**Table 1:** Baseline characteristics of the study populations.

(Hemodialysis patients)		(Controls with normal renal function)	
Characteristic	Value	Characteristic	Value
Male/Female	66/36	Male/Female	27/41
Age, yr	58.5 ± 13.6	Age, yr	58.0 ± 11.5
Principal cause of ESRD		eGFR, mL/min/1.73 m^2^	81 ± 15
Diabetes	34 (33.3)	Diabetes	12 (17.6)
Hypertension	14 (13.7)	Hypertension	34 (50.0)
Glomerulonephritis	26 (25.5)	Hyperlipidemia	31 (45.6)
Polycystic kidney disease	6 (5.9)	Hyperuricemia	4 (5.9)
Other	22 (21.6)		
Medical history		Characteristics in HD group are at 1 year after HD initiation and those in control group are at the time of first ECG examination.	
PCI	5 (4.9)		
Cardiac surgery	2 (2.0)	Values are mean ± SD or n (%).	
Medication		Categorical data are shown as percentages.	
ACEI, ARB, or both	59 (57.8)	Continuous data are presented as means ± standard deviation.	
Beta-blocker	10 (9.8)	ESRD = end-stage renal disease;	
Ca-blocker	54 (52.9)	PCI = percutaneous coronary intervention;	
Digitalis	1 (1.0)	ACEI = angiotensin-converting enzyme inhibitor;	
Other anti-arrhythmic drugs	0 (0.0)	ARB = angiotensin receptor blocker;	
Weight, kg (after dialysis)	57.3 ± 12.6	eGFR = estimated glemerular filtration rate.	
Blood pressure, mmHg			
Systolic	152 ± 23		
Diastolic	83 ± 15		
Cardiothoracic ratio, %	46.8 ± 4.6		
Urea reduction ratio, %	66.0 ± 6.8		
Laboratory data (before dialysis)			
Hemoglobin, g/dl	10.1 ± 1.0		
Albumin, g/dl	3.9 ± 0.3		
Urea, g/dl	68.2 ± 16.5		
Corrected calcium, mg/dl	9.0 ± 0.9		
Phosphorus, mg/dl	5.7 ± 1.4		
Potassium, mEq/l	5.1 ± 0.6		
Magnesium, mg/dl	2.7 ± 0.4		
Glucose, mg/dl	129 ± 45		

### Ventricular repolarization duration and its predictors

Univariate analyses of various variables on QTc interval at 1 year after HD initiation in the HD group are shown in Table 2. The difference in the principal cause of ESRD was found to be associated with QTc interval by one-way layout analysis of variance (*p* = 0.070). QTc interval was found to have a direct relationship with age (*p* = 0.041) and cardiothoracic ratio (*p* = 0.044) and inverse relationship with hemoglobin (*p* = 0.051), corrected calcium (*p* = 0.037) and phosphorus (*p* = 0.033). However, gender, use of ACEIs/ARBs or Ca-blockers, dialysate calcium concentration, and plasma levels of albumin, potassium, magnesium, and glucose did not show any association with QTc interval. The multivariate regression analysis of selected variables (diabetes, age, cardiothoracic ratio, hemoglobin, corrected calcium, and phosphorus) showed that diabetes (*p* = 0.043) and the corrected calcium levels before HD session (*p* = 0.041) were independent predictors of QTc interval prolongation (Table 3).

**Table 2:** Univariate analysis of various variables on QTc interval at 1 year after HD initiation.

Variables	QTc	Regression line	p Value
Gender			0.277
Female	441		
Male	436		
Principal cause of ESRD			0.07
Diabetes	447		
Hypertension	440		
Glomerulonephritis	432		
Polycystic kidney disease	430		
Other	431		
Medication of ACEI, ARB, or both			0.656
Yes	439		
No	436		
Medication of Ca-blocker			0.922
Yes	438		
No	437		
Age		y = 416.225+0.365638x	0.041
Cardiothoracic ratio		y = 384.358+1.12925x	0.044
Hemoglobin		y = 489.496–5.18006x	0.051
Albumin		y = 457.35–5.25164x	0.546
Corrected calcium		y = 489.659–5.83605x	0.037
Phosphorus		y = 459.887–4.005x	0.033
Potassium		y = 469.28–6.37277x	0.128
Magnesium		y = 406.728+8.35133x	0.364
Glucose		y = 430.389+0.0588396x	0.346
Dialysate calcium concentration		y = 469.556–11.0024x	0.372

Unpaired t-test or one-way layout analysis of variance was applied for categorical variables.

Linear regression analysis was applied for continuous variables.

p<0.10 denotes statistical significance.

**Table 3:** Multivariate regression analysis of selected variables on QTc interval at 1 year after HD initiation.

Covariates	Partial regression coefficient	Adjusted p value
Diabetes	10.8821	0.0432
Age	0.155167	0.4447
Cardiothoracic ratio	0.963853	0.1223
Hemoglobin	-0.669074	0.8229
Corrected calcium	-7.24571	0.0414
Phosphorus	-3.17633	0.0875

p<0.05 denotes statistical significance.

### Changes in QTc interval in HD patients and in control patients over several years

**Fig 1:**
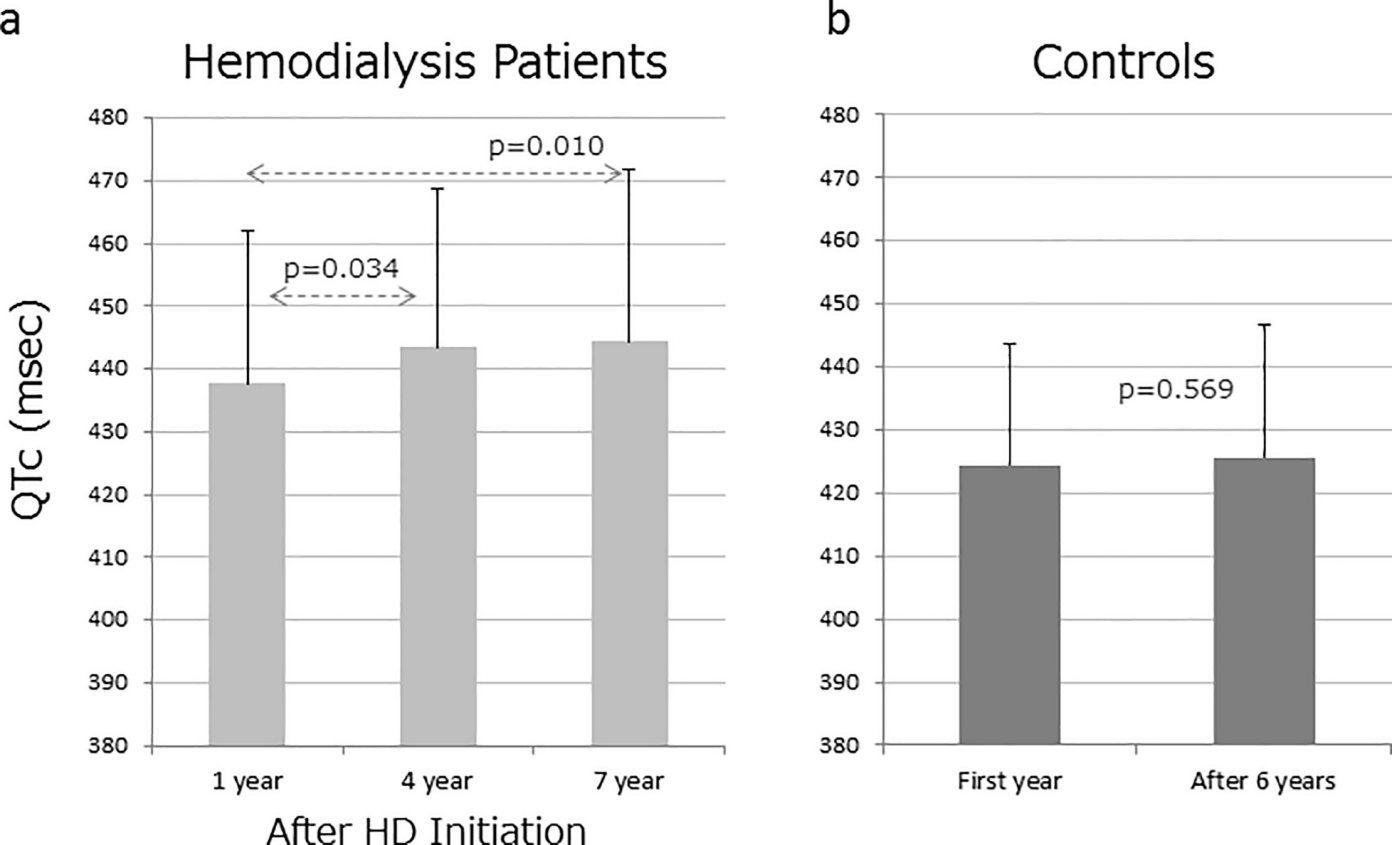
Changes in QTc interval in hemodialysis (HD) group and control group. (A) The QTc interval at 1 year, 4 years, and 7 years after HD initiation in the HD group was compared by Dunett type multiple comparison. (B) For comparison between the QTc interval at first year and after 6 years in the control group, a paired Student’s *t* test was used. The QTc interval at 1 year after HD initiation in the HD group was significantly longer than that at first year in the control group (p<0.001, an unpaired Student’s *t* test).

As shown in Fig 1A, average QTc interval at 1, 4, and 7 years after HD initiation were 437, 443, and 445 ms, respectively. QTc interval at 4 and 7 years after HD initiation were significantly longer (*p*<0.05) than that at 1 year after HD initiation. However, the QTc interval at 4 and 7 years after HD initiation showed no significant difference. On the other hand, average QTc interval at the first year and after an average of 6.3 years in the control group showed no significant difference (425 ms vs 426 ms; *p* = 0.569; Fig 1B), however, it was shorter than that in the HD group (*p*<0.001).

## Discussion

SCD is the leading cause of death in HD patients. Not only coronary artery disease and heart failure which are underlying pathogenic process in the general population, but also factors such as ventricular hypertrophy, electrolyte shift, and vascular calcification, may have important implications for SCD in HD patients [15]. The most common time for SCD to occur is during the last 24 hours of the longer weekend interdialytic period and during the 12 hours after the start of the first dialysis session of the week [16, 17]. SCD from the former is partly accounted for by the increase in fluid, electrolytes, and metabolites, while that from the latter may represent intradialytic risk of arrhythmia and the potential for subsequent fatal event during and immediately after a dialysis session. Recent reports showing the strong relationship between QTc interval and SCD in hypertrophic cardiomyopathy [18, 19] and the impact on QTc interval of vascular calcification which becomes more severe as the stage of CKD progresses [20], allowed us to believe the importance of QTc interval as risk assessment for SCD in HD patients.

We found that the average QTc interval in patients with ESRD at one year after HD initiation was longer as compared to that in patients with normal renal function. A previous study [12] has shown similar results, although the sample size and the way to measure QTc interval (manually vs automatically) were different. Recently, Sherif et al. investigated the impact of various stages of CKD on cardiac repolarization [13]. QTc interval was shown to increase with each successive stage of CKD, while the QTc interval in HD patients was not significantly different as compared to CKD stage 5 patients not on renal replacement therapy [13]. However, in our study, QTc interval at 4 and 7 years after HD initiation were significantly prolonged as compared to that at 1 year after HD initiation. We also found that average QTc interval in control patients did not change in the 6 years, suggesting that the change of QTc interval at 4 to 7 years after HD initiation in CKD patients is not thought to be expected with advancing age. The difference between previous findings [13] and our results can be attributed to differences in methodology and population characteristics. These findings suggest that cardiac repolarization abnormalities are progressive during subsequent stages of CKD and it is difficult for HD therapy to suppress the development of these abnormalities completely.

Reliability of QTc interval is a critical issue to be addressed. To date, numerous studies have utilized QT or QTc interval in drug trials or cardiological investigations. In many of them, QTc interval was divided into two gender-specific categories: normal and prolonged, however the cutoff points were to some extent arbitrary [6, 21–23]. In some papers, the cutoff points for women were 10 to 20 ms longer than those for men [6, 23, 24]. We analyzed QTc interval data as continuous variables without categorizing or gender classification. In the current study, the control group was female-dominant and the HD group was male-dominant. Considering that QTc interval tends to be longer in women than men [25], it does not seem to hinder our conclusion that ventricular repolarization duration was significantly longer in the HD group than in the control group. On the other hand, the two companies that provide most of the ECG machines in Japan apply different computerized algorithms to measure QT interval and different formulae for QTc interval. Although we recalculated the QTc interval data provided by Nihon Kohden to unify the formula into Bazett formula, the difference in algorithm to measure QT interval between the two companies could not be corrected. However, our results regarding the change of QTc interval over several years look more credible, because serial ECG data for each patient was provided by the same company’s machine.

Current analyses of clinically relevant variables on QTc interval at 1 year after HD initiation revealed that the corrected calcium levels before dialysis were a possible predictor of QTc interval prolongation. Several studies have suggested that a low calcium concentration of serum or dialysate and rapid reduction of serum calcium concentrations during HD session may lead to prolongation of QTc interval, possibly resulting in SCD [11, 23, 26]. Alternatively, because serum ionized calcium levels regulate the contractility of cardiac myocytes and vascular smooth muscle, hypocalcemia or rapid lowering of serum calcium levels during dialysis may contribute to hypotension and reduction of coronary blood flow and subsequent fatal myocardial ischemia. Previously, Pun et al reported that patients who received HD with a dialysate concentration of calcium <2.5 mEq/L had a higher risk of SCD than patients treated with higher dialysate calcium concentrations [27]. Although in the present study, no significant difference was seen in QTc interval among patients with different dialysate calcium concentrations (Table 2), dialysate with a higher calcium concentration might be recommended in patients with prolonged QTc intervals, while avoiding hypercalcemia and ectopic calcification.

In the current study, diabetes as a cause of ESRD also has a possible association with QTc prolongation. SCD in patients with diabetes mellitus is known as the “dead in bed” syndrome [28]. Because an independent association between hypoglycemia and QTc prolongation was demonstrated in a large epidemiologic study on type 1 diabetes [22], the “dead in bed” syndrome is believed to be a result of arrhythmia, caused by nocturnal hypoglycemia leading to QT prolongation followed by ventricular tachyarrhythmia. Considering the fact that patients undergoing HD may become hypoglycemic without being aware of it [29], it might be realistic for HD patients, particularly those with diabetes, to have SCD due to the arrhythmogenic effect of QT prolongation by hypoglycemia along with low serum calcium and potassium levels after HD sessions [30].

In conclusion, our study showed that average QTc interval in patients with CKD at 1 year after HD initiation were already long and it was further prolonged with dialysis vintage. In baseline analyses, diabetes and lower plasma calcium levels before dialysis were independently associated with QTc interval prolongation. Thus, close attention to QTc interval during the clinical management of HD patients might be helpful in reducing the mortality due to SCD; however, larger-scale studies are necessary to confirm our results.

## Supporting information

S1 Table Inclusion and exclusion criteria in hemodialysis patients. (PDF)

S2 Table Details of multivariate regression analysis of selected variables on QTc interval at 1 year after HD initiation. (PDF)

S3 Table Source data on which Fig 1 is based. (PDF)
