# Supplementary material for: Changes in QTc interval in long-term hemodialysis patients
Source: PLoS One. 2019 Jan 3;14(1):e0209297. doi: 10.1371/journal.pone.0209297 (PMC6317809; doi:10.1371/journal.pone.0209297)
Supplement: S1 Table — (PDF) [file pone.0209297.s001.pdf]

S1 Table. Inclusion and exclusion criteria in hemodialysis patients.

Inclusion criteria

- Patients receiving HD in outpatient clinics
- Patients who were 25 years or older at the time of HD initiation
- Patients who had been undergoing 4-hour long HD, 3 times a week for at least 7 years
- Patients whose ECG data at 1, 4, and 7 years after the start of HD treatment are available

Exclusion criteria

- Patients with at least one of the following findings on their any ECG at 1, 4, or 7 years:
    - a) Heart rates <57 or >103 bpm
    - b) Any rhythm other than sinus
    - c) Any instances of extrasystoles
