# Supplementary material for: Changes in QTc interval in long-term hemodialysis patients
Source: PLoS One. 2019 Jan 3;14(1):e0209297. doi: 10.1371/journal.pone.0209297 (PMC6317809; doi:10.1371/journal.pone.0209297)
Supplement: S2 Table — (PDF) [file pone.0209297.s002.pdf]

S2 Table. Details of multivariate regression analysis of selected variables on QTc interval at 1 year after HD initiation.

Covariates	Partial regression coefficient	Standard partial regression coefficient	Partial correlation coefficient	Variance inflation	Partial F value	P value
Diabetes	10.8821	0.202627	0.216057	1.04806	4.21111	0.043201
Age	0.155167	0.082299	0.0825156	1.23492	0.589574	0.444687
Cardiothoracic ratio	0.963853	0.174775	0.16595	1.34822	2.43547	0.122291
Hemoglobin	−0.669074	−0.026245	−0.0242019	1.46903	0.0504025	0.822897
Corrected calcium	−7.24571	−0.20183	−0.217933	1.02113	4.28824	0.0413749
Phosphorus	−3.17633	−0.175056	−0.183238	1.1025	2.98787	0.0874791
