# Supplementary material for: Changes in QTc interval in long-term hemodialysis patients
Source: PLoS One. 2019 Jan 3;14(1):e0209297. doi: 10.1371/journal.pone.0209297 (PMC6317809; doi:10.1371/journal.pone.0209297)
Supplement: S3 Table — (PDF) [file pone.0209297.s003.pdf]

S3 Table. Source data on which Fig 1 is based.

(A)

| Patient | 1 year | 4 year | 7 year |
|---|---|---|---|
| HD1 | 468 | 426 | 451 |
| HD2 | 410 | 428 | 425 |
| HD3 | 443 | 481 | 469 |
| HD4 | 439 | 430 | 426 |
| HD5 | 440 | 473 | 465 |
| HD6 | 416 | 436 | 459 |
| HD7 | 435 | 443 | 443 |
| HD8 | 462 | 474 | 470 |
| HD9 | 445 | 481 | 466 |
| HD10 | 418 | 466 | 441 |
| HD11 | 433 | 433 | 433 |
| HD12 | 397 | 418 | 396 |
| HD13 | 441 | 449 | 447 |
| HD14 | 478 | 473 | 477 |
| HD15 | 443 | 440 | 435 |
| HD16 | 457 | 469 | 467 |
| HD17 | 436 | 435 | 427 |
| HD18 | 468 | 449 | 426 |
| HD19 | 435 | 451 | 450 |
| HD20 | 423 | 442 | 435 |
| HD21 | 438 | 457 | 447 |
| HD22 | 461 | 456 | 462 |
| HD23 | 418 | 423 | 437 |
| HD24 | 461 | 458 | 458 |
| HD25 | 493 | 461 | 482 |
| HD26 | 448 | 450 | 433 |
| HD27 | 423 | 428 | 455 |
| HD28 | 436 | 428 | 438 |
| HD29 | 434 | 420 | 474 |
| HD30 | 436 | 501 | 401 |
| HD31 | 426 | 435 | 434 |
| HD32 | 398 | 409 | 410 |
| HD33 | 458 | 429 | 425 |
| HD34 | 406 | 422 | 404 |
| HD35 | 449 | 431 | 418 |
| HD36 | 452 | 472 | 504 |
| HD37 | 455 | 396 | 421 |
| HD38 | 391 | 411 | 394 |
| HD39 | 431 | 441 | 433 |
| HD40 | 380 | 373 | 405 |
| HD41 | 418 | 451 | 391 |
| HD42 | 436 | 452 | 453 |
| HD43 | 482 | 406 | 411 |
| HD44 | 407 | 442 | 438 |
| HD45 | 411 | 416 | 440 |
| HD46 | 436 | 430 | 442 |
| HD47 | 425 | 431 | 378 |
| HD48 | 451 | 418 | 442 |
| HD49 | 405 | 440 | 422 |
| HD50 | 438 | 454 | 509 |
| HD51 | 377 | 393 | 401 |
| HD52 | 440 | 424 | 445 |
| HD53 | 432 | 437 | 431 |
| HD54 | 468 | 485 | 502 |
| HD55 | 408 | 413 | 432 |
| HD56 | 439 | 460 | 455 |
| HD57 | 465 | 485 | 469 |
| HD58 | 448 | 442 | 425 |
| HD59 | 410 | 413 | 520 |
| HD60 | 434 | 445 | 468 |
| HD61 | 461 | 448 | 445 |
| HD62 | 422 | 444 | 440 |
| HD63 | 437 | 442 | 438 |
| HD64 | 466 | 448 | 426 |
| HD65 | 434 | 444 | 446 |
| HD66 | 452 | 514 | 456 |
| HD67 | 434 | 433 | 450 |
| HD68 | 431 | 428 | 444 |
| HD69 | 413 | 496 | 444 |
| HD70 | 445 | 423 | 437 |
| HD71 | 466 | 464 | 475 |
| HD72 | 482 | 482 | 502 |
| HD73 | 488 | 479 | 492 |
| HD74 | 431 | 442 | 426 |
| HD75 | 465 | 475 | 487 |
| HD76 | 464 | 447 | 437 |
| HD77 | 396 | 405 | 401 |
| HD78 | 436 | 434 | 466 |
| HD79 | 464 | 466 | 461 |
| HD80 | 449 | 442 | 441 |
| HD81 | 400 | 430 | 416 |
| HD82 | 382 | 402 | 382 |
| HD83 | 453 | 428 | 430 |
| HD84 | 433 | 416 | 431 |
| HD85 | 473 | 480 | 487 |
| HD86 | 436 | 428 | 432 |
| HD87 | 455 | 440 | 470 |
| HD88 | 447 | 432 | 425 |
| HD89 | 440 | 437 | 434 |
| HD90 | 412 | 425 | 444 |
| HD91 | 463 | 441 | 473 |
| HD92 | 400 | 416 | 444 |
| HD93 | 428 | 447 | 424 |
| HD94 | 439 | 454 | 448 |
| HD95 | 472 | 506 | 455 |
| HD96 | 401 | 422 | 441 |
| HD97 | 421 | 434 | 477 |
| HD98 | 465 | 450 | 458 |
| HD99 | 417 | 441 | 448 |
| HD100 | 462 | 461 | 461 |
| HD101 | 459 | 479 | 481 |
| HD102 | 433 | 470 | 445 |

(B)

| Patient | First year | After 6 years |
|---|---|---|
| CTRL1 | 446 | 453 |
| CTRL2 | 416 | 436 |
| CTRL3 | 408 | 404 |
| CTRL4 | 402 | 418 |
| CTRL5 | 410 | 417 |
| CTRL6 | 435 | 436 |
| CTRL7 | 402 | 378 |
| CTRL8 | 420 | 431 |
| CTRL9 | 443 | 454 |
| CTRL10 | 433 | 442 |
| CTRL11 | 430 | 424 |
| CTRL12 | 435 | 441 |
| CTRL13 | 429 | 406 |
| CTRL14 | 404 | 385 |
| CTRL15 | 442 | 434 |
| CTRL16 | 430 | 436 |
| CTRL17 | 390 | 412 |
| CTRL18 | 443 | 453 |
| CTRL19 | 452 | 426 |
| CTRL20 | 401 | 419 |
| CTRL21 | 418 | 414 |
| CTRL22 | 395 | 398 |
| CTRL23 | 441 | 438 |
| CTRL24 | 450 | 443 |
| CTRL25 | 418 | 417 |
| CTRL26 | 401 | 416 |
| CTRL27 | 431 | 413 |
| CTRL28 | 420 | 401 |
| CTRL29 | 398 | 415 |
| CTRL30 | 437 | 458 |
| CTRL31 | 432 | 385 |
| CTRL32 | 444 | 447 |
| CTRL33 | 447 | 433 |
| CTRL34 | 420 | 411 |
| CTRL35 | 431 | 460 |
| CTRL36 | 395 | 403 |
| CTRL37 | 440 | 430 |
| CTRL38 | 404 | 415 |
| CTRL39 | 435 | 451 |
| CTRL40 | 452 | 449 |
| CTRL41 | 411 | 444 |
| CTRL42 | 453 | 443 |
| CTRL43 | 400 | 402 |
| CTRL44 | 422 | 448 |
| CTRL45 | 453 | 465 |
| CTRL46 | 396 | 412 |
| CTRL47 | 450 | 453 |
| CTRL48 | 368 | 392 |
| CTRL49 | 429 | 428 |
| CTRL50 | 407 | 395 |
| CTRL51 | 441 | 450 |
| CTRL52 | 436 | 422 |
| CTRL53 | 446 | 454 |
| CTRL54 | 434 | 450 |
| CTRL55 | 436 | 403 |
| CTRL56 | 418 | 433 |
| CTRL57 | 417 | 420 |
| CTRL58 | 423 | 409 |
| CTRL59 | 409 | 398 |
| CTRL60 | 425 | 422 |
| CTRL61 | 418 | 419 |
| CTRL62 | 432 | 443 |
| CTRL63 | 419 | 422 |
| CTRL64 | 449 | 429 |
| CTRL65 | 425 | 399 |
| CTRL66 | 397 | 407 |
| CTRL67 | 453 | 462 |
| CTRL68 | 419 | 416 |
